# Why children are not vaccinated against measles: a cross-sectional study in two Nigerian States

**DOI:** 10.1186/2049-3258-72-48

**Published:** 2014-12-29

**Authors:** Anne Cockcroft, Muhammad U Usman, Obrian F Nyamucherera, Henry Emori, Bong Duke, Nisser Ali Umar, Neil Andersson

**Affiliations:** CIET Trust Botswana, Gaborone, Botswana; Ministry of Health, Bauchi State Nigeria; CIET Trust, 71 Oxford Road, Saxonwold, Johannesburg 2196 South Africa; State Planning Commission, Cross River State Government, Cross River State Nigeria; Primary Health Care Development Agency, Bauchi State Nigeria; CIET-PRAM, Department of Family Medicine, McGill University, Montreal, Canada; Centro de Investigación de Enfermedades Tropicales, Universidad Autónoma de Guerrero, Acapulco, Mexico

**Keywords:** Vaccination, Measles, Immunisation, Children, MDG4, Nigeria

## Abstract

**Background:**

Childhood vaccination rates in Nigeria are among the lowest in the world and this affects morbidity and mortality rates. A 2011 mixed methods study in two states in Nigeria examined coverage of measles vaccination and reasons for not vaccinating children.

**Methods:**

A household survey covered a stratified random cluster sample of 180 enumeration areas in Bauchi and Cross River States. Cluster-adjusted bivariate and then multivariate analysis examined associations between measles vaccination and potential determinants among children aged 12-23 months, including household socio-economic status, parental knowledge and attitudes about vaccination, and access to vaccination services. Focus groups of parents in the same sites subsequently discussed the survey findings and gave reasons for non-vaccination. A knowledge to action strategy shared findings with stakeholders, including state government, local governments and communities, to stimulate evidence-based actions to increase vaccination rates.

**Results:**

Interviewers collected data on 2,836 children aged 12-23 months in Cross River and 2,421 children in Bauchi. Mothers reported 81.8% of children in Cross River and 42.0% in Bauchi had received measles vaccine. In both states, children were more likely to receive measles vaccine if their mothers thought immunisation worthwhile, if immunisation was discussed in the home, if their mothers had more education, and if they had a birth certificate. In Bauchi, maternal awareness about immunization, mothers’ involvement in deciding about immunization, and fathers’ education increased the chances of vaccination. In Cross River, children from communities with a government immunisation facility were more likely to have received measles vaccine. Focus groups revealed lack of knowledge and negative attitudes about vaccination, and complaints about having to pay for vaccination. Health planners in both states used the findings to support efforts to increase vaccination rates.

**Conclusion:**

Measles vaccination remains sub-optimal, particularly in Bauchi. Efforts to counter negative perceptions about vaccination and to ensure vaccinations are actually provided free may help to increase vaccination rates. Parents need to be made aware that vaccination should be free, including for children without a birth certificate, and vaccination could be an opportunity for issuing birth certificates. The study provides pointers for state level planning to increase vaccination rates.

## Background

An estimated 2.5 million children under five years of age died from vaccine preventable diseases in 2002, with 1.1 million of these deaths in Africa [1]. Effective programmes of childhood vaccination can help to reduce this mortality and to achieve Millennium Development Goal 4 (a two-thirds reduction in mortality rates for children under the age of 5 years between 1990 and 2015) [2]. There have been some international successes attributed to measles vaccination, with a reported global decrease in measles mortality between 2000 and 2007 [3].

UNICEF estimated 84% measles vaccination coverage (among one year old children) globally in 2012, with 42% in Nigeria [4]. The Nigeria Demographic and Health Survey (DHS) of 2013 also reported a coverage of only 42% with measles vaccine nationally [5] and the Nigeria Multiple Indicator Cluster Survey (MICS) in 2011 reported 56% of children aged 12-23 months had received measles vaccine (at any age) [6]. While measles cases fell globally between 2000 and 2011, in the Africa Region the annual measles incidence rose again from a low point in 2008, to 227 per million in 2011, including a large measles outbreak (18,843 cases) in Nigeria in 2011 [7].

The Nigerian national immunisation policy states that government will provide immunisation services and potent vaccines free of charge to all populations at risk of vaccine preventable diseases, with the intention of achieving 90% coverage with all vaccines by 2020 [7]. All states of the Nigerian federation have immunisation strategic plans up to 2015, in line with the MDG targets. The routine childhood vaccination schedule includes five visits. Bacille Calmette-Guerin vaccine (BCG) is given at birth; pentavalent vaccine (diphtheria, pertussis, typhoid, hepatitis B and haemphilus influenzae b) at six, 10 and 14 weeks; oral polio vaccine at birth, six, 10, and 14 weeks; yellow fever vaccine at nine months (right upper arm); and measles vaccine at nine months (left upper arm) [8].

Studies in Nigeria have identified a number of factors related to vaccination among children aged 12-23 months. A study in Northern Nigeria reported that complete non-vaccination was related to factors of maternal knowledge and attitudes, while partial non-vaccination was more related to problems with vaccination services [9]. In a single rural community in Nigeria, maternal knowledge about vaccinations was related to childhood vaccination status [10]. Analyses of data from the 2003 Nigeria Demographic and Health survey found individual socio-economic factors and the community level of hospital deliveries were related to vaccination [11] and explored the reasons for urban children being more likely to be vaccinated [12].

We used a mixed methods approach [13] to examine factors related to measles vaccination and to explore parental reasons for non-vaccination in two states of Nigeria. In 2011, a survey on integrated management of childhood illnesses covered a random sample of communities in Cross River and Bauchi states of Nigeria. We present here the quantitative survey findings about measles vaccination among children age 12-23 months and the factors related to vaccination status, together with qualitative findings from community focus groups that discussed the results of the survey. We also describe how the findings of this mixed methods research are being used to support state level efforts to improve childhood vaccination rates.

## Methods

Between July and September 2011, a cross-sectional household survey enquired about integrated management of childhood illnesses. The survey was part of a programme to support evidence-based planning of health services in two states of Nigeria [14, 15]. The two states are not intended to represent the whole country. Bauchi in the north-east of Nigeria is predominantly Islamic and polygamy is common. Cross River is in the south eastern corner of the country, the main religion is Christianity, and families are typically more nuclear.

The survey covered a random cluster sample of enumeration areas (EAs) from the 2006 Nigerian national census, stratified by urban/rural location. The sample included 90 EAs in each of the two states, including 10 EAs in each of three focus Local Government Authorities (LGAs) per state. Within each sample EA, the cluster comprised contiguous households radiating from a random starting point and continuing until data were collected from mothers or caregivers of 100-150 children under four years old. All children under four years old in the households were included, unless there was no caregiver present to answer questions about the child.

A questionnaire administered by trained interviewers to mothers or caregivers of children aged less than four years old asked about immunisation and care and treatment of childhood illnesses. Field workers recorded the measles vaccination status of children, as reported by the mother or main caregiver. Field workers described the measles vaccination using a phrase in the local language meaning “the injection into the left upper arm at nine months” to assist mothers’ recall. Pre-testing confirmed this was well-understood by mothers. We did not verify the mothers’ reports by checking vaccination cards among those who held them. The questionnaire also asked about mothers’ education, and decision-making, knowledge and attitudes about vaccination. A question about birth registration of the child was included because of interest in this in its own right, as well as the possibility that it might be related to vaccination status. The field teams also collected information from each household on their demographics and socio-economic status and recorded information from key informants in each community about access to health services.

Three months after the household survey, field teams returned to the same 90 communities in each state to conduct separate male and female focus groups to feedback and to discuss findings from the household survey. Each discussion lasted for about one hour with 8-12 participants drawn from adults with young children in the community. For every group there was a facilitator and a reporter who took detailed notes. The groups discussed why parents did not take their children for immunisation and suggested how parents could be encouraged to take their children for immunisation. The facilitators and reporters together finalized the reports of the discussions. Two of the authors conducted thematic analysis of the reports from the groups to identify common themes in the discussions and extracted relevant quotes. In this qualitative analysis we did not count how many focus groups mentioned each theme, but rather continued searching for new themes in the group reports until no further new themes emerged.

### Analysis

Data entry relied on EpiInfo version 6 [16] and employed double data entry with validation to minimize keystroke errors. Analysis relied on CIETmap open source software which provides a user friendly interface with the R statistical language [17]. Analysis weighed all estimates proportional to the population, by rural/urban location, and allowing for the over-sampling in the three focus LGAs in each state.

We examined the association of potential determinants with measles vaccination status among children aged 12-23 months: factors related to the individual child; indicators of household socio-economic status; maternal empowerment; parental education, knowledge and attitudes about immunisation; and community factors. Because of very different educational levels between the two states (Table 1), we defined “more education” as being any formal education in Bauchi and as being junior secondary education of higher in Cross River.

**Table 1:** **Vaccination status and potential determinants among children aged 12-23 months**

	Cross river state	Bauchi state
Number of children in the study	2836	2421
	n/N (weighted %)	n/N (weighted %)
Received measles vaccination	2332/2790 (81.8)	1049/2387 (42.0)
*Child factors*		
Sex (male)	1427/2836 (50.8)	1221/2420 (51.2)
With birth certificate	1400/2782 (48.4)	446/2389 (19.1)
*Household socio-economic status*		
Household considers their financial situation to be average or above	1805/2823 (64.7)	1918/2406 (80.8)
Household had enough food in the last week	2262/2815 (81.2)	2176/2406 (89.3)
Father with higher paying occupation	1211/2830 (42.7)	490/2385 (20.4)
Female headed household	428/2831 (15.2)	15/2410 (0.6)
*Maternal empowerment*		
Mother has an income of her own and takes part in the decision how to spend it	1243/2680 (47.0)	1250/2405 (51)
Mother or caregiver decided about immunising the child	1995/2754 (73.8)	184/2361 (8.1)
*Parental education*		
Mother with any formal education	2647/2815 (94.1)	453/2420 (18.0)
Father with any formal education	2556/2691 (95.5)	841/2390 (33.8)
Mother with junior secondary or higher education	1739/2815 (62.9)	205/2420 (8.8)
Father with junior secondary or higher education	1928/2691 (72.5)	625/2390 (25.7)
*Maternal knowledge and attitudes*		
Mother has heard about immunisation	2544/2563 (99.0)	2154/2341 (90.5)
Mother thinks immunising children is worthwhile	2555/2601 (98.0)	1997/2383 (83.8)
Household discussed immunisation at home	2224/2594 (85.6)	1777/2377 (74.2)
*Community factors*		
Urban community	953/2836 (32.4)	406/2421 (19.5)
Community has an active village health committee	1479/2670 (56.3)	584/2402 (17.9)
Community received immunisation campaign visits apart from polio days	1711/2542 (66.6)	643/2312 (29.1)
Community has a government health facility providing immunisation services	1601/2771 (57.1)	1226/2412 (50.2)

We analysed the findings from the two states separately. There is no intention that the two states together represent the situation in the whole of Nigeria, and the overall project under which the survey was conducted focuses on supporting evidence-based health planning at state level [14, 15]. In each state separately, we examined the association between measles vaccination and potentially associated variables first in bivariate analysis and then in multivariate analysis using the Mantel Haenszel procedure [18] adjusted for clustering [19]. To allow for variables operating at different levels, we applied a cluster adjustment with a non-fixed OR; with large datasets from stratified random cluster samples this produces findings similar to those of generalised linear mixed model (GLMM) analysis [19]. In the multivariate analysis we began with a saturated model including all the variables significantly associated with measles vaccination in the bivariate analysis. We then used a step-down approach to reach the final model including only variables that remained significantly associated with measles vaccination. We describe associations using the odds ratio (OR) with the cluster adjusted 95% confidence interval (CI).

### Ethical approval

We obtained formal ethical approval for the conduct of the study from the Ministry of Health in each state. The individual field team leaders sought consent for the survey from leaders in each community, while interviewers sought verbal consent from the head of each household as well as from each individual respondent. Interviewers did not record any names or identifying information for households or individuals, and were trained not to proceed with any interview unless they could do so without being overheard.

## Results

For 14,017 children under four years old identified in Cross River State, 12.2% (1790) of mothers or caregivers were not available for interview and 0.7% (117) refused the interview. For 12,154 children under four years old identified in Bauchi State, 1.7% (192) of mothers were unavailable and 0.4% (48) refused. More women work outside the home in Cross River than in Bauchi. Among the total of children aged under four years old, the field teams collected information from mothers and caregivers of 2,836 children aged 12-23 months in Cross River State and 2,421 such children in Bauchi State.

Table 1 shows the measles vaccination status of children aged 12 – 23 months in each state and the distribution of potential determinants of vaccination in several groups: factors specific to the individual child; indicators of household socio-economic status; maternal empowerment variables; parental education; maternal knowledge and attitudes about immunisation; and community factors. The measles vaccination rate was 81.8% (95% CI 80.4-83.3) in Cross River, and much lower in Bauchi at 42.0% (95% CI 40.0-44.0). There were differences between the two states in the levels of several factors. In Cross River the majority of mothers and fathers of the children had at least junior secondary education, while in Bauchi only a minority had any formal education. The mother of the child was rarely involved in the decision about immunisation in Bauchi, but most mothers were involved in this decision in Cross River. About half of children in Cross River had a birth certificate, but only a fifth of those in Bauchi had a certificate.

The educational levels of the mothers in our sample in the two states are similar to those reported among women aged 15-49 years in the 2013 DHS, which reported no education in 73% of women in Bauchi and in 9% of women in Cross River [5].

Table 2 shows the bivariate associations between potential determinants and measles vaccination status. Some factors were significantly associated with measles vaccination in both states: more educated parents, possession of a birth certificate, discussion of immunisation in the household, and mothers who thought immunising children was important. In addition, in Cross River, children of mothers with an income of their own and discretion about spending it and those from communities with a government health facility providing immunisation were more likely to have had measles vaccination. In Bauchi state, children of mothers who had heard of immunisation and those whose caregiver decided about immunisation were more likely to have had measles vaccination.

**Table 2:** **Bivariate analysis of factors related to measles vaccination in children 12-23 months old**

Factor	Cross river	Bauchi
	OR (95% CIca)	OR (95% CIca)
*Child factors*		
Sex (male)	0.96 (0.79-1.15)	1.04 (0.88-1.22)
With birth certificate	**2.74 (2.16-3.47)**	**3.35 (2.50-4.50)**
*Household socio-economic status*		
Household considers their financial situation to be average or above	**1.42 (1.15-1.75)**	1.29 (0.99-1.69)
Household had enough food in the last week	**1.57 (1.26-1.96)**	1.06 (0.76-1.48)
Father has higher paying occupation	1.12 (0.87-1.45)	**2.05 (1.58-2.65)**
Female headed household	0.87 (0.67-1.12)	1.16 (0.44-3.04)
*Maternal empowerment*		
Mother has an income of her own and takes part in decision on how to spend it	**1.31 (1.07-1.62)**	0.94 (0.76-1.16)
Mother or caregiver decided about immunizing the child	0.87 (0.69-1.08)	**2.11 (1.47-3.01)**
*Parental education*		
Mother has any formal education		**3.71 (2.74-5.05)**
Father has any formal education		**2.94 (2.20-3.91)**
Mother has junior secondary or higher education	**1.90 (1.51-2.39)**	
Father has junior secondary or higher education	**1.56 (1.26-1.94)**	
*Maternal knowledge and attitudes*		
Mother has heard about immunisation	1.32 (0.35-4.99)	**8.59 (4.69-15.72)**
Mother thinks immunising children is worthwhile	**2.91 (1.48-5.72)**	**7.56 (5.20-10.98)**
Household discussed immunisation at home	**2.80 (2.11-3.72)**	**5.00 (3.76-6.65)**
*Community factors*		
Urban community	1.22 (0.87-1.70)	**2.04 (1.32-3.16)**
Community has an active village health committee	1.17 (0.85-1.62)	**1.61 (1.04-2.51)**
Community received immunization campaign visits apart from polio days	1.19 (0.80-1.75)	1.38 (0.92-2.06)
Community has a government health facility providing immunization services	**1.54 (1.12-2.11)**	**1.48 (1.00-2.18)**

Bold font indicates bivariate associations significant at the 5% level.

Tables 3(a) and (b) show the final multivariate models of variables associated with measles vaccination in the two states. Among child-specific factors, possession of a birth certificate was relevant. A child with a birth certificate was more than twice as likely to have received measles vaccination in both states. The indicators of household socio-economic status did not remain significantly associated with vaccination status. Maternal empowerment remained important in Bauchi: if the mother decided about immunising the child, that child was more likely to have received measles vaccination. Factors of parental education, knowledge and attitudes were more prominent in Bauchi. In both states the mother’s view that immunisation was worthwhile, and discussion in the home about immunisation remained significantly associated with measles vaccination status. In Cross River only, one community factor remained significantly associated with measles vaccination: the presence of a government health facility offering vaccination within the community.

**Table 3:** **Final model for multivariate analysis of variables associated with measles vaccination status among children aged 12-23 months**

(a) In cross river state	
Factor	OR (95% CIca)
Child has birth certificate	2.62 (1.98 - 3.27)
Mother thinks immunising children is worthwhile	2.73 (1.15 - 4.31)
Household discussed about immunisation at home	2.51 (1.78 - 3.24)
Community has a government health facility providing immunisation services	1.64 (1.17 - 2.12)
**(b) In bauchi state**	
**Factor**	**OR (95% CIca)**
Child has birth certificate	2.29 (1.68 - 2.90)
Mother or caregiver decides about immunising the child	1.89 (1.18 - 2.61)
Mother has any formal education	1.94 (1.39 - 2.49)
Father has any formal education	1.73 (1.26 - 2.20)
Mother has heard about immunisation	3.87 (1.68 - 6.06)
Mother thinks immunising children is worthwhile	3.40 (1.80 - 5.00)
Households discussed about immunisation at home	2.41 (1.58 - 3.25)

The multivariate analysis used the Mantel Haenszel procedure [17] adjusted for clustering [18].

### Views from the focus group discussions

#### Reasons why children are not vaccinated

An emerging theme was that childhood vaccination has to compete with other activities in the household. Other activities, such as farming work, may be considered more important than taking time to have children immunised.

“Our women don’t have the time to stand in a queue and wait for their turn to immunise their children. They have to go to the farm and attend to other household activities.” (Cross River, male group)

Another theme concerned negligence or lack of education, particularly among women.

“It is due to negligence that some women don’t take their children for immunisation or they don’t complete the course.” (Bauchi, male group)

“Illiteracy is the cause of people not immunizing their children (Cross River, male group)

It emerged that some parents consider vaccination to be unnecessary or ineffective.

“There is no difference seen between those children that are immunised and those that are not immunised.” (Bauchi, female group)

A common general theme was that vaccination may do more harm than good. Many groups blamed side effects from vaccination, such as fever and local soreness, as a reason for parents not taking children for vaccination. Others described fears and misconceptions about vaccination, such as that vaccination would lead to infertility or even death of their children. Some described conspiracy theories around vaccination.

“Some people are afraid of the side-effects. When they see another child with any side-effects, they become afraid and don’t take their own children for immunisation” (Bauchi, female group).

“The government has a hidden agenda on immunisation. I think like this because there are so many other diseases that need assistance but they only talk about childhood immunisation. Look at what happened in Kano. They were testing the CSM vaccine and killed so many children. Therefore, some people believe it is not safe”. (Bauchi, male group)

Another common general theme concerned problems with vaccination services. Groups in both states complained of difficult access to facilities or lack of vaccines at facilities.

“The health facility is very far away. That is why we only go once or twice”. (Bauchi, female group).

“We have to go three to four times. Each time we go, health workers tell us that they don’t have vaccines at the health facility. We have become tired of this, which is why we don’t bother going there anymore”. (Bauchi, female group).

Participants complained that health workers demanded payments for vaccination which they know is supposed to be free.

“It is because immunisation workers collect money from our wives. Since we don’t have money to give to the health workers at the immunisation centres, our wives don’t go for immunisation”. (Cross River, male group)

A particular theme emerged related to birth registration. Some groups mentioned that women did not immunise their children because they had delivered them at home and had not registered their births, and they believed that children without a birth certificate cannot be vaccinated in government health facilities.

“We think that when we deliver at home the child cannot be immunized in the hospital”. (Cross River, female group)

#### Suggestions on how to increase vaccination

Suggestions for increasing vaccination levels mirrored the identified reasons for parents not vaccinating their children. People called for better access to services and for vaccination to be provided free of charge.

“Health facilities should be brought closer to the people”. (Cross River, male Group)

“If the vaccines are always available, people can go at any time and receive the immunisation for their children”. (Bauchi female group)

“Government should make sure that immunisation is free”. (Cross River, female group)

Suggestions for suitable channels for encouraging vaccination included community and religious leaders, women leaders, health workers and mass media campaigns.

“The Chief will tell them and they will hear” (Cross River, female group)

“Religious leaders (Imam) can convince people to immunise their children”. (Bauchi, female group)

“Health workers should tell people that if a child is fully immunised, he or she will be more healthy and active than those not immunised”. (Bauchi, female group)

Some of the groups in Bauchi felt it was important first to convince the husband, who could then convince his wife, or allow her to take the child for vaccination.

“The Sarki should educate husbands on the importance of it, so that our husbands in turn will allow us to immunise our children”. (Bauchi, female group)

“A husband can enlighten and convince his wife on the importance of vaccination”. (Bauchi, male group)

## Discussion

The population weighted vaccination coverage of children aged 12-23 months was 81.2% in Cross River and 41.3% in Bauchi. These figures are higher than coverage reported by the 2011 MICS, with its smaller sample in each state: 69.4% in Cross River and 35.7% in Bauchi [6]. Our survey in 2011 shows higher coverage than the 2013 DHS: 77.1% in Cross River and 20.3% in Bauchi [5]. Both the MICS and the DHS surveys estimated vaccination coverage based on vaccination cards (when these were available) or on maternal recall when no card was available. Coverage estimates from the two sources were similar. In the MICS survey, just 29% of children aged 12-23 months had a card available [6].

Reports for the World Health Organisation in 2009 reviewed peer-reviewed publications and grey literature describing reasons for non-vaccination in low and middle income countries [20, 21]. Geographic barriers to access and missed opportunities to vaccinate were a consistent theme, but reasons for non-vaccination related to parental knowledge or attitudes were more region-specific and may reflect underlying health seeking behaviours and perceptions [20]. The review noted that factors linked to under-vaccination were mostly related to vaccination services, while those linked to complete non-vaccination were more often related to parental knowledge and attitudes. A similar finding was reported from a study in northern Nigeria [9]. Our analysis found parental education, knowledge and attitudes were associated with measles vaccination status and, in Cross River, access to facilities offering vaccination was associated with measles vaccination.

In both Bauchi and Cross River, parental attitudes and beliefs were associated with childhood measles vaccination. Children whose mothers thought childhood vaccination was worthwhile were more than twice as likely to have received measles vaccine. This fits with the focus group findings which suggested that some children were not vaccinated because their parents did not think there was any need to vaccinate them, or were concerned about side-effects or suspicious about adverse effects of vaccination. They considered the risks of vaccination outweighed the possible benefits. Other studies in Africa have described an association between parents’ attitudes about immunisation and knowledge of its protective value and immunisation of their children [9, 20, 22]. We also found that children with more educated mothers were more likely to have received measles vaccine. Other studies in low and middle income countries and in sub-Saharan Africa have reported an association between maternal education and childhood vaccination [20, 23].

Discussion about vaccination in the home was associated with measles vaccination in children in both states in our study. We have previously demonstrated in a randomised controlled trial in Pakistan that stimulating evidence-based community *discussions* about vaccination can increase vaccination rates, even in the absence of any supply-side improvements [24]. A behaviour change model (CASCADA) that expands the Knowledge-Attitudes-Practice model places discussion as the immediate precursor to action (in this case, vaccination of the child) [25–27].

In both Bauchi and Cross River, children with a birth certificate were at least twice as likely to have received measles vaccine. This association has not previously been reported in Nigeria. Possession of a birth certificate is not a requirement for access to free vaccination in Nigeria [8], so it is unlikely that children without a birth certificate were actually refused free vaccination. However, the focus group discussions confirmed that some parents *believe* that children without a birth certificate are not entitled to free vaccination, and therefore may not take children to be vaccinated if they do not have a birth certificate. The video docudrama produced to share the study findings and stimulate actions included a scene where a mother says she cannot take her child to be vaccinated because she does not have a birth certificate and this misconception is corrected by another character. The association between possession of a birth certificate and being vaccinated could also be because careful parents both register their child’s birth and take the child for vaccination. Closer links between vital registration services and health services, including taking the opportunity to register children when they are brought for vaccination, have been proposed as a method of increasing birth registration in Africa [28, 29]; a case study from Ghana attributed increased birth registration to promoting links between registration and health services [30]. In Bauchi and Cross River there has been no particular campaign to provide birth registration for children at the time of vaccination, so it is not likely that the association between birth certification and measles vaccination is because children had their birth registered when they were taken for vaccination.

There were some differences between the two states in the associations with vaccination status. For effective planning to improve vaccination rates, it is important to identify local factors related to non-vaccination. These may differ between regions and countries [20] and between different regions of the same country [31]. In Bauchi, father’s education was associated with increased measles vaccination, but this was not the case in Cross River. The findings from the focus groups helped to clarify this. Groups in Bauchi specifically mentioned the need for the father’s consent and support for children to be taken for vaccination; perhaps such support is more forthcoming from more educated fathers. Also in Bauchi only, children of mothers who were more aware and knowledgeable about vaccination were more likely to be vaccinated. A previous study in a rural area of Nigeria found maternal knowledge about vaccination to be an important determinant of vaccination [10] and a small trial in Karachi reported increased vaccination rates among children of poorly-literate mothers after an educational intervention [32]. A randomised controlled trial in southern Pakistan showed a clear increase in uptake of immunisation following discussion of the costs and benefits [24].

In Bauchi, female empowerment had an effect, such that children whose caregivers (usually the mother) were involved in deciding about vaccination were more likely to have received measles vaccine. This was not a factor in Cross River, where maternal education levels were notably higher than in Bauchi. Other authors have reported that when women have decision making autonomy their children were more likely to be fully immunised [20].

In Cross River state, children from communities with a government health facility providing vaccination services were more likely to have received measles vaccine. This was not a significant factor in Bauchi, where vaccination rates were lower overall. Poor access to vaccination services has frequently been reported as a reason for non-vaccination of children [20].

### Use of the findings by State Governments

We shared the study results with stakeholders in both states. A score card listed the levels of key indicators for each LGA, with a state average for comparison; a docudrama portrayed evidence from the survey and invited discussions of community led solutions. Field teams used the docudrama to conduct structured discussions with gender stratified groups of community representatives and leaders, and with pregnant women, their spouses, and families. At State and LGA level we used the score cards, and the docudrama and discussion guide, to discuss findings with the Executive Governor, and with ministries, line departments, and other organisations, and to support action plans to improve access to and quality of health services. The discussions in both states were timed to link with the annual budget and planning process.

Evidence about vaccination generated from the survey has been used by the Bauchi and Cross River state governments. The ministry of budget and planning in Bauchi State coordinated the 2012 and 2013 budgets so that the evidence could be used in developing the state strategic plan. The Primary Health Care Development Agency used the survey findings to plan their 2013 annual expenditure. They used the evidence to ask policy makers to allocate more resources to address the low rate of childhood vaccination, and successfully defended their budget requests using the findings from the survey. In Cross River State, the State Planning Commission collaborated with the local government authorities to use the evidence from the survey as their baseline data for performance measurement in their Local Government Economic Empowerment and Development Strategy (LEEDS) for the period 2013-2016.

The surveys reported here are parts of a programme to support evidence-based health planning in the two states [15]. Several factors have contributed to the successful uptake of the findings: the topics for the surveys in the programme are chosen by the state governments; state governments, especially the health ministries, are closely involved in design, implementation and analysis of the findings; the surveys are timed to coincide with the planning and budgeting cycle; and the findings are disaggregated to Local Government Authority level.

### Limitations

As with all cross-sectional studies, we can only describe associations between the outcome and potential determinants; we cannot draw definite conclusions about causality. We did not collect information about other factors potentially related to vaccination status, such as religion or ethnicity. We relied on maternal recall of measles vaccination status. In developed countries, some authors concluded that maternal recall is not as good an indicator of vaccination status as health facility records [33, 34], but others concluded it was as good as vaccination cards [35, 36].

A study in India found that maternal recall underestimated children’s vaccination status, but vaccination cards were not helpful because less than half the mothers had cards and they were often incomplete or grossly inaccurate [37]. In Egypt, mothers’ reports were later confirmed by card data for at least 83% of children [38]. Studies in Costa Rica and Sudan concluded that maternal recall was good enough for estimating vaccination status, especially for single dose vaccines [39, 40]. Authors from Guatemala highlighted serious problems with service-based data (including vaccination cards) [41]. Even if maternal recall may under- or over-estimate vaccination status, this is not related to factors such as maternal education or poverty status [37, 42]. We therefore believe that our reliance on maternal recall of vaccination status is reasonable and is not likely to have introduced bias into the analysis of factors related to vaccination in the two states.

## Conclusions

Coverage of measles vaccination remains sub-optimal in both states, particularly in Bauchi. Efforts to counter negative perceptions about vaccination and to ensure vaccinations are actually provided free of charge may help to increase vaccination rates. This in turn will reduce the costs of illness transferred to already poor families in the event of a measles epidemic [43]. Parents need to be made aware that vaccination should be provided free of charge, including for children without a birth certificate, and vaccination could be an opportunity for registering children without birth certificates. The study provides pointers for state level planning to increase vaccination rates.

## Abbreviations

95% CI: 95% confidence interval
DHS: Demographic and health survey
LGA: Local Government Authority
MDG: Millennium development goal
MICS: Multiple indicator cluster survey
OR: Odds ratio.
